# Biological Effect of Thiazole-Containing Zinc(II) Phthalocyanine on Different Sizes of Gold Nanoparticles

**DOI:** 10.1021/acsomega.4c08762

**Published:** 2025-07-03

**Authors:** Nazlı Farajzadeh Öztürk, Hilal Zengin Uzunmehmetoğlu, Hacer Yasemin Yenilmez, Sadin Özdemir, Abdurrahman Dündar, Zehra Altuntaş Bayır

**Affiliations:** † Department of Analytical Chemistry, Faculty of Pharmacy, Acıbadem Mehmet Ali Aydınlar University, Ataşehir, TR-34752 Istanbul, Turkey; ‡ Department of Chemistry, 52971 Istanbul Technical University, Maslak, TR-34469 Istanbul, Turkey; § Food Processing Programme, Technical Science Vocational School, 52983 Mersin University, Yenisehir, TR-33343 Mersin, Turkey; ∥ Department of Medical Services and Techniques, Vocational School of Health Services, Mardin Artuklu University, TR-47420 Mardin, Turkey

## Abstract

This study aims to design multidisciplinary bioagents for a wide range of biological applications. The synthesis and characterization of 4,5-bis­((4-phenylthiazol-2-yl)­thio)­phthalonitrile (**a**) and its octa-substituted zinc­(II) phthalocyanine derivative (**b**) were described in this study. Additionally, gold nanoparticles were synthesized in three different sizes, including 10 nm (**1**), 45 nm (**2**), and 80 nm (**3**). Macromolecule (**b**) was used for surficial functionalization of gold nanoparticles (**1–3**) to prepare nanoconjugates (**1–3b**). Antioxidant, antimicrobial, antibacterial, antibiofilm, antidiabetic, and deoxyribonucleic acid (DNA) cleavage activities of biocandidates (**b**, **1–3**, and **1–3b**) were examined to determine the optimum size of gold nanoparticles and the effect of modifying groups on their bioactivity in this study for the first time. The highest antioxidant activities were obtained for biocandidates (**b** and **1b**) at 100 mg/L. The best minimum inhibitory concentration (MIC) values were obtained at 32 mg/L for bioagents (**b**, **1**, and **3b**) against *E*. *faecalis* whereas the MIC value was obtained at 32 mg/L for **1b** against *E*. *hirae* and *E*. *faecalis*. Bioagents (**b** and **1–3b**) exhibited high APDT activities (16 mg/L) against the studied microorganisms. The highest biofilm inhibition activities were obtained 94.57 and 89.28% for 50 mg/L nanoconjugate (**1b**) against *S*. *aureus* and *P*. *aeruginosa*, respectively. All the studied biocandidates inhibited 100% *E*. *coli* viability at 50 mg/L. The antidiabetic activities of biocandidates (**b**, **1–3**, and **1–3b**) were obtained between 7.52 and 100 mg/L. Bioagents (**2**, **3**, **1b**, and **2b**) destroyed the DNA integrity, as well. The significant improvement in the biological activities of gold nanoparticles confirmed that new nanoconjugates especially **1b** can be considered promising medical nanomaterials after further clinical investigation.

## Graphical abstract

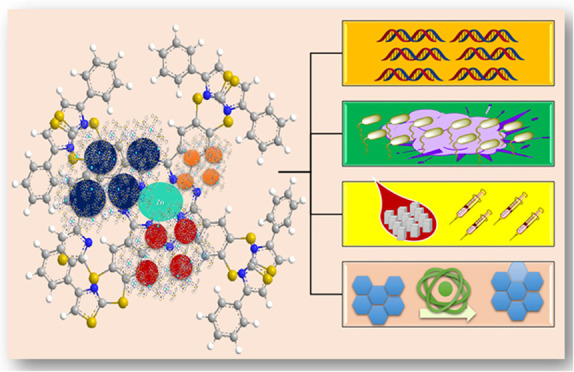

## Introduction

Nanotechnology is a developing high-tech field encompassing synthesis, functionalization, and characterization of a wide range of metallic, organic, and inorganic materials at the nanoscale (1–100 nm).^1,2^ Among noble metallic nanostructures (Pt, Ag, Au, Pd), gold nanoparticles display astonishing properties originating from quantum size and large surface-to-volume ratio owing to their flexible characteristics.^3^ Some studies have reported their potential utility in diverse scientific applications such as electronics, food packaging, sensors, and photonics.^4–6^ Particularly, colloidal gold solution has been utilized for the treatment of mental disorders and syphilis owing to its curative features along with amazing biological stability for many years.^7,8^ Due to the biocompatibility and nontoxic nature of gold nanoparticles, they have been considered amazing bioagents for gene and drug delivery along with (bio)­medical applications.^9^ Recognition of biological interactions occurring between gold nanoparticles and biological systems at the nanobiointerface is one of the essential factors for the appropriate utility of these nanoparticles. These interactions can be often managed by size and surficial modification.^10,11^ The probable ease of their size-controlled synthesis followed by the position of plasmon bands makes them excellent tunable agents for many biological fields such as clinical therapeutics, biological sensing, and imaging. Besides, the surficial functionalization of gold nanoparticles can lead to the alteration of surface chemistry which is a vital parameter for diverse criteria encompassing surface charge, malignant membrane modality, cell uptake, and cytotoxicity.^11^ So far, the surface of gold nanoparticles has been modified with various functional groups consisting of polymers, deoksiriboz nükleik asit (DNA)-binding drugs, antibodies, and heterocycles.^12–15^ Jaswal et al. used polydopamine (PDA)-modified gold nanospheres (GNSs) for the fabrication of polycaprolactone (PCL). The resultant nanofibrous composites (PCL-GNSs@PDA) were applied for photothermal bone cancer therapy and bone regeneration using MC3T3-E1 cell lines. They demonstrated potent cure properties and adhesive performances for the tested cancer tissues.^16^ Xie et al. prepared 4,6-diamino-2-pyrimidinethiol (DAPT)-functionalized gold nanoparticles (DAPT-GNPs, DGNPs) using different sizes of gold nanoparticles (2–14 nm) and studied their antibacterial properties. The ultrasmall-modified gold nanoparticles exhibited the highest antibacterial activities.^17^ In our previous study, two new octa-substituted metal phthalocyanines bearing 4,5-bis­(4-(dimethylamino)­phenyl)­ethynyl were synthesized and used for the functionalization of gold nanoparticles. The newly synthesized nanoconjugates displayed higher biological activities than those of the phthalocyanines and unmodified gold nanoparticles.^18^ Although some studies have presented the improving effect of phthalocyanines on the individual features of gold nanoparticles in the past decade, there is an unignorable vacancy in the design of bioactive phthalocyanine-modified gold nanoparticles in the literature.^19,20^

Phthalocyanines are N-heterocyclic macromolecules including a highly π-conjugated ring. This planar aromatic ring demonstrates high electron transfers correlating to outstanding electrical and physical features as well as thermal and chemical stability.^21–23^ However, the low solubility of phthalocyanines in a wide range of organic and aqueous media is a basic drawback limiting their applications in numerous scientific fields and technologies. Interestingly, the structural flexibility of phthalocyanine rings is an important characteristic that not only improves their solubility but also makes possible the dedication of different biological, chemical, and optical properties through the addition of certain bulky substituents on the phthalocyanine periphery or/and the placement metal ions into the ring center.^24–29^ Albeit a vast range of long or bulky groups can be utilized for peripheral alteration of phthalocyanine ring, heterocyclic compounds consisting of carbazoles, imidazoles, triazoles, and thiazoles have been used to design proper phthalocyanine-based agents for medical, electrochemical, physical, and industrial applications in recent years.^30–32^ Particularly, thiazole-containing phthalocyanines exhibited excellent pharmaceutical and biological properties like antimicrobial, anticancer, and anti-inflammatory activities.^33–35^

Thiazoles play a basic role in the formation of numerous natural compounds like chlorophyll, vitamins, hemoglobin, DNA, and RNA. Due to the planarity and aromaticity of the C_3_H_3_NS ring, they display high π-electron delocalization leading to wonderful physiological functions. The high chemical adaptability of the thiazole derivatives can provide different requirements for biological systems.^36–38^ Hydrogen peroxide, hydroxyl radical, superoxide, peroxyl radical, and hypochlorous acid are a series of biologically vital oxygen-derived compounds that damage lipids, proteins, and DNA molecules. A suitable antioxidant is defined as a substance whose low amount is sufficient for the oxidation of large oxidizable species. Antioxidants can inhibit lipid peroxidation in foods. Also, they can prevent or delay the damage of free radicals or oxidants inducing the fundamental degradation of living cells.^39^ Recently, the antioxidant activity of some thiazole-based compounds has been investigated extensively in the literature.^40^ Generally, thiazoles can interfere with DNA synthesis through weak interactions such as hydrogen bonds, coordination bonds, and π-stacking interactions and subsequently arrest cell growth and division. The anticancer properties of thiazole-based agents that can be appropriate alternatives for traditional drugs have been proven in the literature.^33,40^ Besides, bacteria, viruses, and fungi are pathogenic microorganisms that can result in infectious diseases. These disorders threaten human health and even lead to deathful results worldwide.^41–43^ Although many medicinal methods and pharmaceutical compounds have been discovered to fight these microorganisms, the challenge to design new alternatives continues owing to the increasing diversity of pathogens, mutation of microbes, and bacterial resistance. As thiazoles exhibit amazing biological activities, they have taken an essential position in drug design. On the other hand, side effects of anticancer and antimicrobial drugs are vital drawbacks that make scientists probe or design new more efficient biological approaches or bioactive materials not including faults of the conventional ones.^44,45^ Photosensitive molecules can be activated after exposure to light and result in the generation of reversible oxygen species required for photodynamic anticancer and antimicrobial therapies.^46^ Also, antimicrobial photodynamic therapy (APDT) is considered a low systemic toxic method that has much fewer noninvasive side effects and leads to the eradication of pathogenic microorganisms and the reduction/elimination of bacterial resistance.^47–50^ Recently, phthalocyanines have attracted remarkable attention since their biological properties can be modified by structural changes (substituents and metal ions). The combination of phthalocyanine and thiazole compounds can unify the individual biological properties to overcome various disadvantages of currently available clinical drugs or to develop novel efficient multidisciplinary ones.^33^

There are only a few literature reports on the synthesis and characterization of phthalocyanine-containing nanomaterials, especially phthalocyanine/metal-based nanoconjugates, for biological applications. On the other hand, the optimum size of nanostructures for biological activities has not been studied extensively in the literature. This study presents a new disubstituted phthalonitrile derivative and its zinc­(II) phthalocyanine bearing thiazole groups. The newly synthesized phthalocyanine was used to modify gold nanoparticles of three different sizes to improve their characteristics. For the first time, the biological properties of the resultant phthalocyanine-gold nanoconjugates were studied extensively to optimize the size of bioactive gold nanoparticles. To the best of our knowledge, the effect of the gold nanoparticles’ size on the biological features was investigated for the first time in this study by the examination of the 2,2-diphenyl-1-picrylhydrazyl (DPPH) radical scavenging, antimicrobial, antimicrobial photodynamic therapy (APDT), antidiabetic, microbial cell viability inhibition, biofilm inhibition, and DNA cleavage activities of the unmodified and modified gold nanoparticles.

## Results and Discussion

### Synthesis and Characterization

The synthetic routes for 4,5-bis­((4-phenylthiazol-2-yl)­thio)­phthalonitrile (**a**) and its octa-substituted zinc­(II) phthalocyanine derivative (**b**) are portrayed in Scheme 1. Compound (**a**) resulted from the combination of 4,5-dichlorophthalonitrile and 4-phenylthiazole-2-thiol via the aromatic substitution reaction occurring between chlorine atoms of the phthalonitrile and thiol groups of the substituents. Pure compound (**a**) was obtained by recrystallization from ethanol. Characterization of compound (**a**) was carried out by applying ^1^H NMR, ^13^C NMR, and FT-IR spectroscopic techniques. All the results were in accordance with the predicted structure. Macromolecule (**b**) was synthesized by cyclotetramerization of compound (**a**) in the presence of zinc­(II) acetate and characterized by performing ^1^H NMR, FT-IR, UV–vis, and MALDI-TOF mass spectroscopy. All the data was assigned to the target macrocyclic complex.

**Scheme 1:**
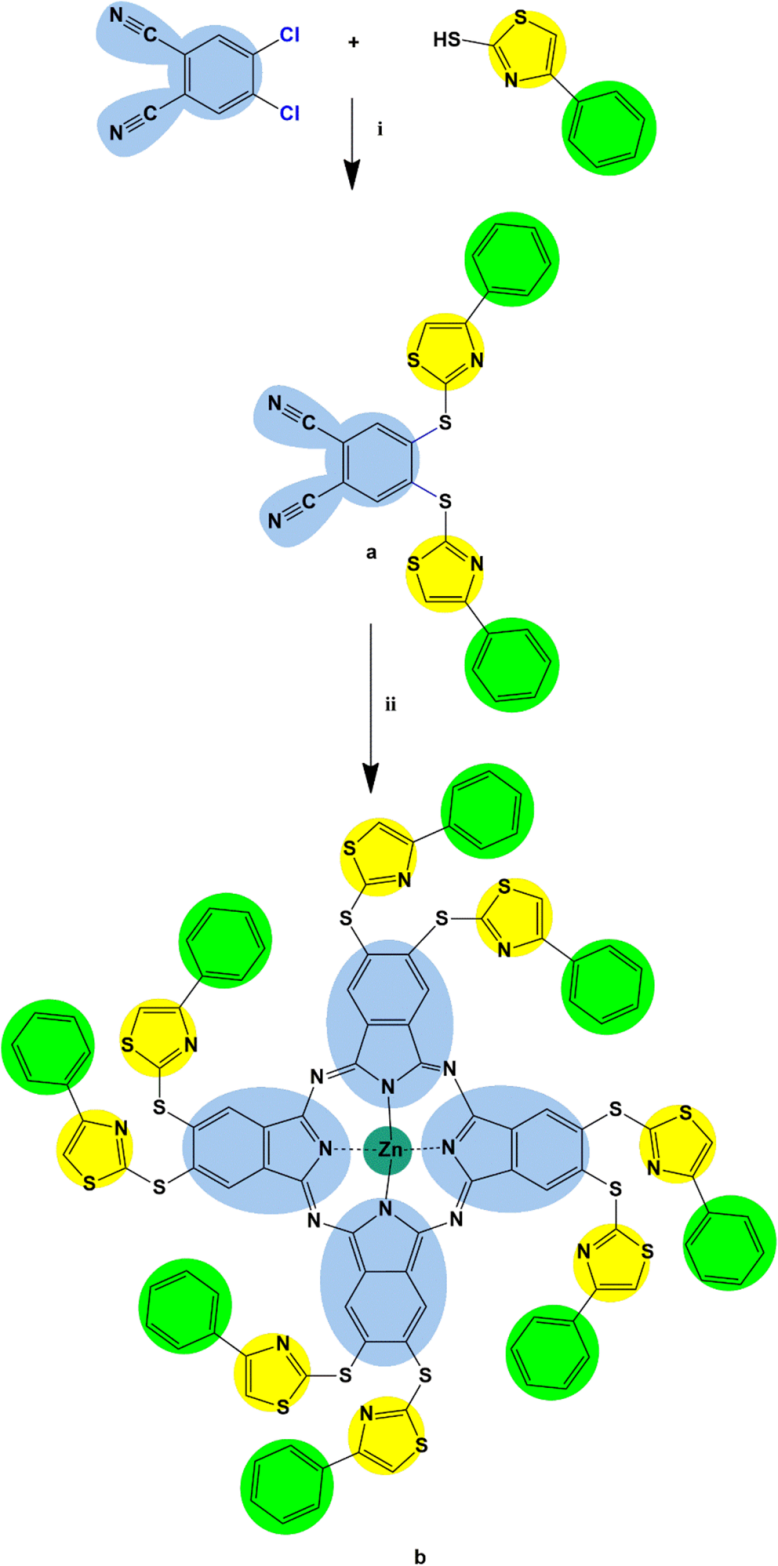
Synthetic Procedure for Compounds (**a** and **b**)­^a^

a (i) Dry dimethyl sulfoxide, potassium carbonate, 80 °C, 48 h; (ii) zinc­(II) acetate, *N*, *N*-dimethylaminoethanol, 135 °C, 24 h.

Gold nanoparticles (**1–3**) differing in size (10, 45, and 80 nm) were synthesized using various chloroauric acid/trisodium citrate ratios. As the size of gold nanoparticles increased, the SPR band shifted to longer wavelengths. The SPR bands of gold nanoparticles (**1–3**) were observed at 517, 523, and 530 nm, respectively. Nanoconjugates (**1–3b**) were prepared by surficial modification of gold nanoparticles (**1–3**) with macromolecule (**b**). Figure 1 demonstrates the TEM images of nanoconjugates (**1–3b**). Generally, modified gold nanoparticles (**1–3b**) became much closer to each other owing to nonbonding interactions occurring between gold nanoparticles (**1–3**) and macromolecule (**b**) as well as π-π interactions between the phthalocyanine rings. Figure 2 portrays the morphological characteristics of gold nanoparticles (**1**) after modification with compound **b**.^51^ The FESEM image of modified gold nanoparticles (**1b**) confirms the successful coverage of the surface of gold nanoparticles (**1**) with compound **b** (Figure 2a,b). The elements presented in modified gold nanoparticles (**1b**) are depicted in the EDS map (Figure 2c). The elemental mapping analysis indicated the presence of carbon, sulfur, nitrogen, zinc, gold, and oxygen elements distributed uniformly in nanobioagent **1b**.^52^ The results proved that the surface of gold nanoparticles (**1**) was successfully modified by compound **b** through the nonbonded interactions. The zeta potential of the unconjugated (**1–3**) and phthalocyanine-functionalized gold nanoparticles (**1–3b**) were also measured. The respective zeta potentials of unmodified gold nanoparticles (**1–3**) were obtained −53.1 ± 1.3, −44.8 ± 1.3, and −28.0 ± 1.3 mV whereas those of the modified gold nanoparticles (**1–3b**) were obtained −34.3 ± 1.7, −31.7 ± 0.7, and −17.9 ± 0.8 mV, respectively. The zeta potentials of unmodified gold nanoparticles were negative owing to the presence of negatively charged citrate groups and inversely proportional to the size of gold nanoparticles. Moreover, the linkage of the zinc phthalocyanine to the surface of gold nanoparticles mainly via nonbonding interactions (e.g., electrostatic interactions) increased the zeta potentials of all the nanoconjugates.^53^ As the size of gold nanoparticles decreased, more citrate groups were placed on the surface which led to more surficial modification with the phthalocyanine and more difference in the zeta potentials of the unmodified certain-sized gold nanoparticles and the related nanoconjugate. As expected, the highest difference was obtained for zeta potentials of the smallest gold nanoparticles (**1**) and its nanoconjugate (**1b**). In the FT-IR spectra of the modified gold nanoparticles, the intensity of the C=O peaks appearing around 1700 cm^–1^decreased owing to nonbonding interactions between citrate groups and the zinc­(II) phthalocyanine after conjugation.^54^ Additionally, the characteristic peaks of the phthalocyanine were observed in the FT-IR spectra of gold nanoparticles. The FT-IR spectra of gold nanoparticles (**1**) and nanoconjugate (**1b**) are portrayed in the “Supporting Information’’ as an example.

**Figure 1:**
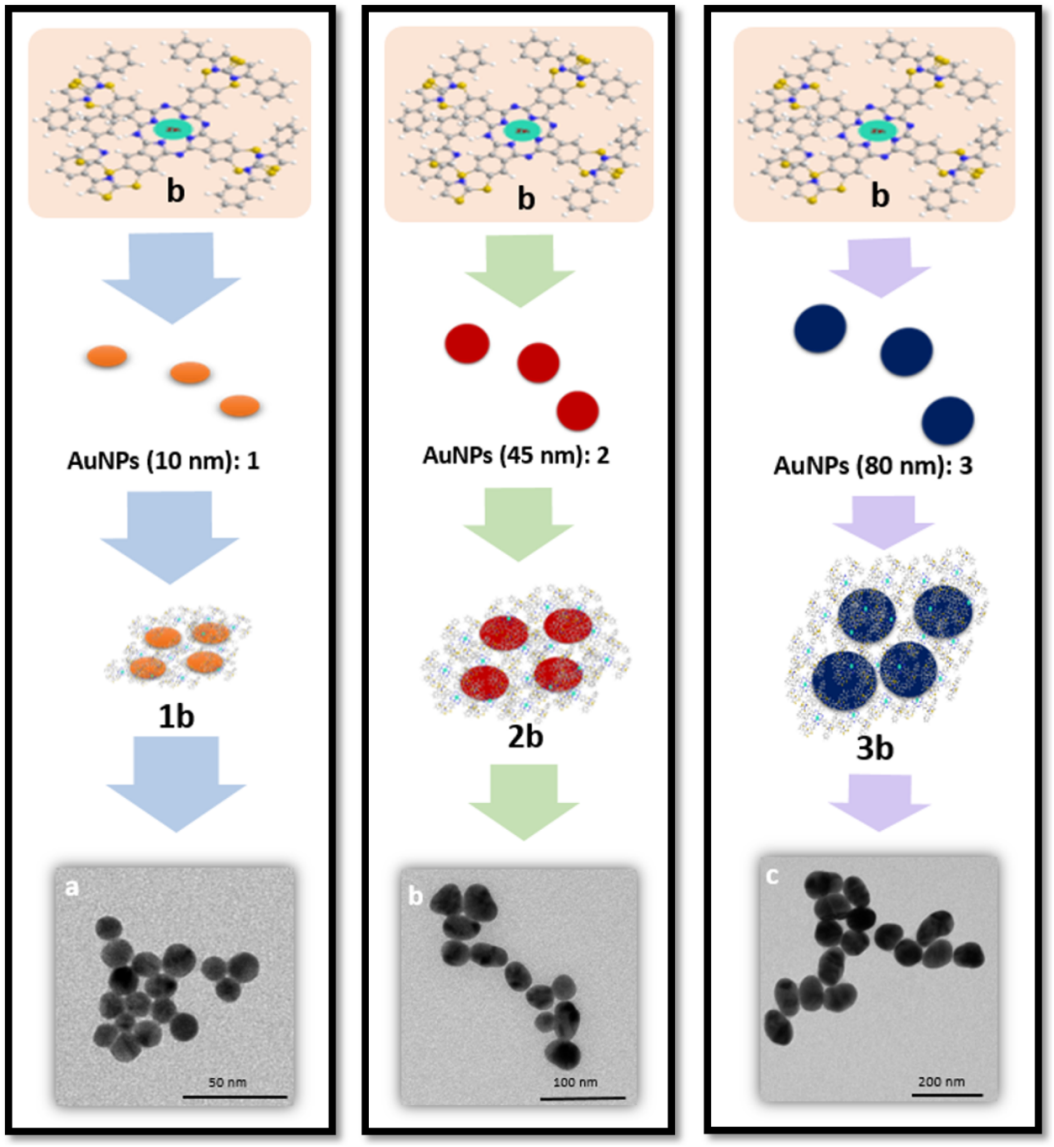
TEM images of nanoconjugates **1b** (AuNPs; 10 nm/ZnPc), **2b** (AuNPs; 45 nm/ZnPc), and **3b** (AuNPs; 80 nm/ZnPc).

**Figure 2:**
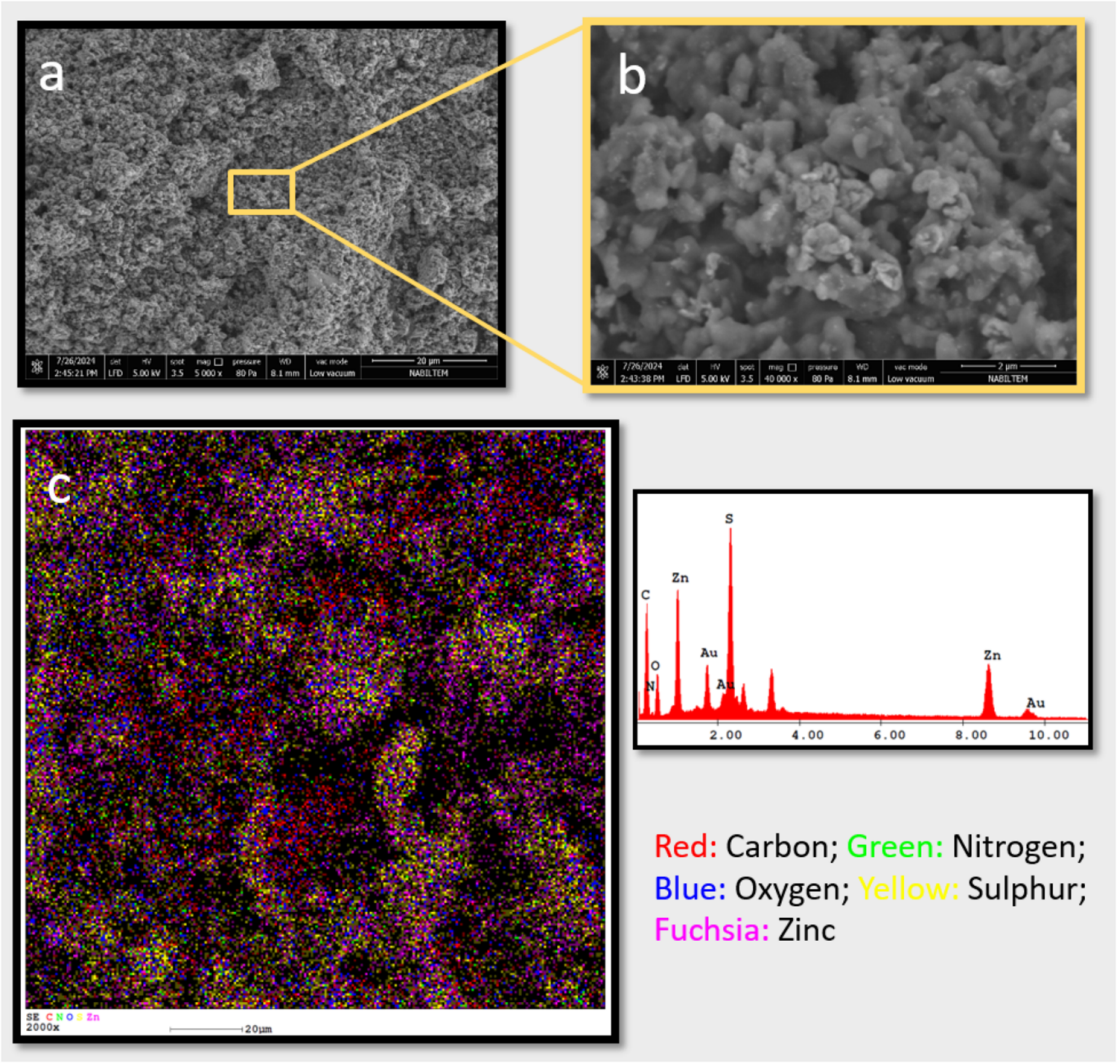
(a,b) The FESEM image of modified gold nanoparticles (**1b**). (c) The elemental mapping analysis of modified gold nanoparticles (**1b**).

### Biological Studies

#### DPPH Scavenging Ability

DPPH free radical scavenging assay is a rapid and concise method that reveals the antioxidant potentials of biological candidates and is often preferred by researchers.^55,56^ Antioxidants may reduce the risk of diverse diseases like heart disease and some cancers. They scavenge free radicals from body cells, thus preventing or reducing the damage that the oxidized molecules can cause to biomolecules such as proteins, enzymes, and DNA. In this study, the DPPH scavenging activities of biocandidates (**b**, **1–3**, and **1–3b**) were investigated at different concentrations with erosion-release mechanisms (Figure 3). Accordingly, it was observed that the antioxidant activities of all the studied samples enhanced as the concentration increased. The highest activity was obtained for macromolecule (**b**) at different concentrations ranging from 6.25 (23.58 ± 1.26%) and 100 mg/L (73.29 ± 3.94%). The respective antioxidant activities of gold nanoparticles (**1–3**) were obtained 48.54 ± 2.62, 44.56 ± 2.43, and 40.00 ± 2.25% whereas those of nanoconjugates (**1–3b**) were obtained 68.04 ± 3.53, 64.63 ± 3.43, and 61.52 ± 3.36% at 100 mg/L, respectively. The antioxidant activities of 100 mg/L biocandidates (**b**, **1–3**, and **1–3b**) decreased in the order of: **b** > **1b** > **2b** > **3b** > **1** > **2** > **3**. Since an increase in the size of gold nanoparticles led to a decrease in the antioxidant activity, there was an inverse correlation between the size and antioxidant activity. Additionally, surficial modification of gold nanoparticles (**1–3**) with macromolecule (**b**) significantly refined their antioxidant activities. Some studies have presented the antioxidant activities of various phthalocyanine derivatives and nanostructures. Günsel et al. studied the DPPH scavenging activity of 2(3), 9(10), 16(17), 23(24)-tetrakis (4-(dimethylamino) benzyloxy) zinc­(II) phthalocyanine at 500 μg/mL. The antioxidant activity of the related phthalocyanine was obtained 20%.^55^ Aydın et al. obtained 50% antioxidant activity for new tetra-substituted zinc­(II) phthalocyanines bearing 4-(methylthio)­phenylthioxy groups on peripheral positions.^57^ Aghamirzaei et al. prepared biologically gold nanoparticles using Chinese lettuce leaf extracts and determined their DPPH scavenging activity. The antioxidant activities were obtained between 40.66 and 77% at different concentrations ranging from 20 to 100 mg/L.^58^ The antioxidant potential of phthalocyanines is directly linked to the resonance of localized electrons. The bonded substituent and the centrally located metal atom have a direct effect on the p electron density. As a result, biocandidates (**b**, **1–3**, and **1–3b**) can be utilized as antioxidant agents after clinical research.

**Figure 3:**
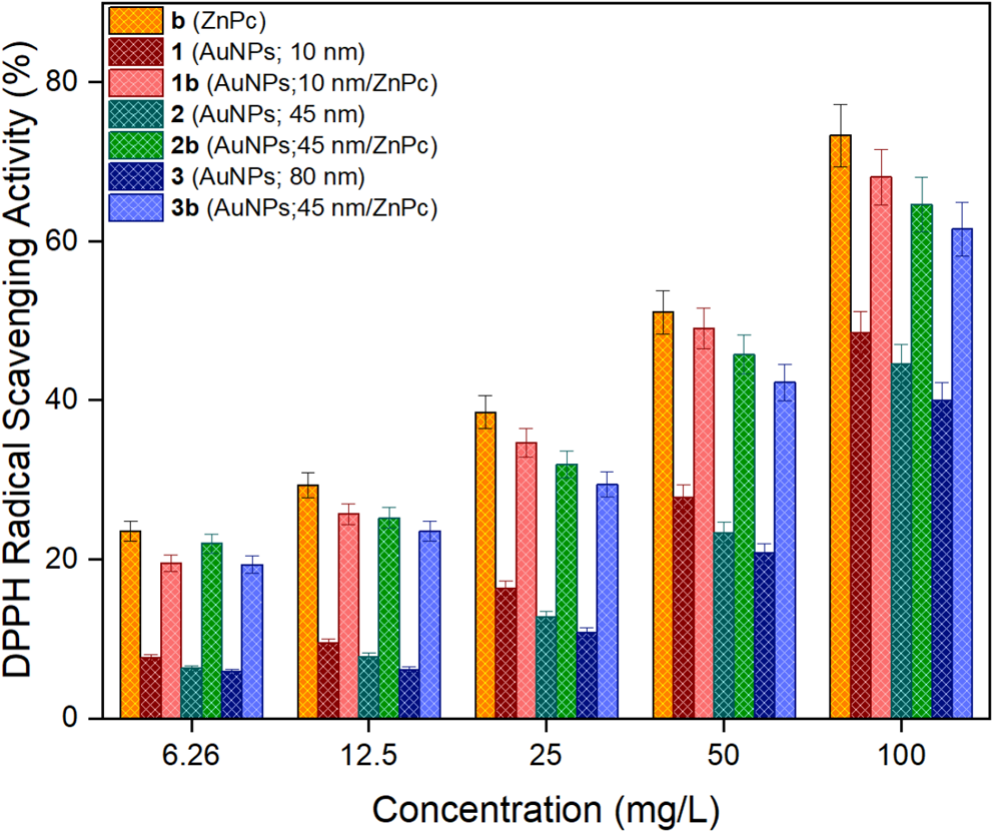
DPPH scavenging activities of biocandidates **b** (ZnPc), **1** (AuNPs; 10 nm), **2** (AuNPs; 45 nm), **3** (AuNPs; 80 nm), **1b** (AuNPs; 10 nm/ZnPc), **2b** (AuNPs; 45 nm/ZnPc), and **3b** (AuNPs; 80 nm/ZnPc).

### Antimicrobial and Photodynamic Antimicrobial Therapy Activity

Perhaps one of the most important problems of today and the coming years is the resistance that microorganisms have developed against antibiotics. The antimicrobial potential of synthetic or natural molecules, which many scientists are working on, is an important issue. In this study, the antimicrobial activities of biocandidates (**b**, **1–3**, and **1–3b**) were examined against some bacteria and yeasts exhibiting its effect with diffusion release mechanism. The determined MIC values are listed in Table 1. All the studied biocandidates were more effective against Gram-positive bacteria. Accordingly, *E*. *feacalis* was the most sensitive microorganism for all the tested biocandidates. The best MIC value was obtained 32 mg/L for biocandidates (**b**, **1**, and **2b)** against *E*. *faecalis* whereas the same MIC value was determined for nanoconjugate (**1b)** against two different microorganisms (*E*. *hirae* and *E*. *faecalis*). Additionally, the MIC values of nanoconjugate (**1b)** were obtained 64, 128, 64, 64, 128, and 128 mg/L against *E*. *coli*; *P*. *aeruginosa*; *L*. *pneumophila* subsp. *pneumophila*; *S*. *aureus*; *C*. *albicans*; and *C*. *tropicalis*, respectively. As a result, nanoconjugate (**1b)** displayed the highest antimicrobial activities. Albeit surficial functionalization of gold nanoparticles (**1–3**) with macromolecule (**b**) improved the antimicrobial activities, the size of gold nanoparticles did not affect their antimicrobial activities significantly.

**Table 1:** Antimicrobial Activities of Biocandidates **b** (ZnPc), **1** (AuNPs; 10 nm), **2** (AuNPs; 45 nm), **3** (AuNPs; 80 nm), **1b** (AuNPs; 10 nm/ZnPc), **2b** (AuNPs; 45 nm/ZnPc), and **3b** (AuNPs; 80 nm/ZnPc)

		MIC values (mg/L)												
microorganisms		**b**		**1**		**1b**		**2**		**2b**		**3**		**3b**
*E. coli*		128		128		64		128		64		128		128
*P. aeruginosa*		128		128		128		128		64		128		64
*L. pneumophila* subsp. *pneumophila*		64		128		64		128		128		128		64
*E. hirae*		64		64		32		64		64		64		64
*E. faecalis*		32		32		32		64		32		64		64
*S. aureus*		64		64		64		64		64		64		64
*C. albicans*		128		128		128		256		128		256		128
*C. tropicalis*		128		256		128		256		128		256		256

In this study, antimicrobial photodynamic activities (APDT) of biocandidates (**b**, **1–3**, and **1–3b**) were also tested. The MIC values are given in Table 2. All the studied biocandidates exhibited higher antimicrobial activities (2 or 4 times) after irradiation. The MIC value was obtained 16 mg/L for macromolecule (**b**, **1b**, **2b**, and **3b**) against *E*. *hirae* and *E*. *faecalis*; *E*. *hirae*; *E*. *faecalis* and *S*. *aureus*; and *L*. *pneumophila*. Nanoconjugate (**1b**) demonstrated the highest APDT activity. Additionally, the respective MIC values of nanoconjugate (**1b**) were obtained 32, 64, 32, 32, 32, 64, and 64 mg/L against *E. coli*; *P. aeruginosa*; *L. pneumophila* subsp. p*neumophila*; *E*. *feacalis*; *S*. *aureus*, *C*. *albicans*, and *C*. *tropicalis*. Although the exposure to light did not affect the antimicrobial activities of gold nanoparticles (**1–3**) significantly, light-activation of nanoconjugates (**1–3b**) improved their antimicrobial activities. There are some similar studies in the literature. Magadla and Nyokong studied the antimicrobial activities of some zinc­(II) phthalocyanines-doped silica nanoparticles against *S*. *aureus* with and without irradiation. The studied phthalocyanine-based nanoconjugate killed 15% of the microorganism at 45 mg/L in the dark; however, some of them induced a significant decrease in *S*. *aureus* growth significantly decreased with irradiation at 660 nm for different periods (15–60 min). The exposure time did not influence the antibacterial activities and a 15 min period was considered an ideal time for irradiation.^59^ Aghamirzaei et al. also stated good antimicrobial activity for biologically synthesized gold nanoparticles against *S*. *aureus* and *P*. *aeruginosa*.^58^ Compared to the literature, biocandidates (**b**, **1–3**, and **1–3b**) exhibited highly effective antimicrobial activities. More further investigations are required to ensure their efficiency as antimicrobial agents against a wide range of microorganisms.

**Table 2:** APDT Activities of Biocandidates **b** (ZnPc), **1** (AuNPs; 10 nm), **2** (AuNPs; 45 nm), **3** (AuNPs; 80 nm), **1b** (AuNPs; 10 nm/ZnPc), **2b** (AuNPs; 45 nm/ZnPc), and **3b** (AuNPs; 80 nm/ZnPc)

		MIC values (mg/L)												
microorganisms		**b**		**1**		**1b**		**2**		**2b**		**3**		**3b**
*E. coli*		32		64		32		64		32		128		64
*P. aeruginosa*		64		128		64		128		32		128		32
*L. pneumophila* subsp. *pneumophila*		32		64		32		128		64		128		16
*E. hirae*		16		32		16		64		32		64		32
*E. faecalis*		16		32		32		64		16		64		32
*S. aureus*		32		64		32		64		16		64		32
*C. albicans*		64		128		64		128		64		256		64
*C. tropicalis*		64		256		64		256		64		256		128

### Biofilm Inhibition Activity

The survival of microorganisms under different environmental conditions and their potent formation of surface-associated biofilms are well-known as essential pathogenicity factors. The building blocks of biofilms are extracellular polymeric substances (EPS) in which biofilm microorganisms are embedded.^60^ Biofilm formation, considered an industrial problem for many years, plays a vital role in many healthcare-associated infections, especially foreign body infections, and contributes to the pathogenesis of infections by allowing pathogens to evade antimicrobial drugs and the host’s immune response.^61^ In this study, the effect of biocandidates (**b**, **1–3**, and **1–3b**) on the inhibition of exopolysaccharide biofilm produced by *S*. *aureus* and *P*. *aeruginosa* bacteria were examined at different concentrations (12.5, 25, and 50 mg/L) (Figures 4 and 5). All the biocandidates were released via erosion. The increase in concentration was followed by the enhancement in the biofilm inhibition activities of all the biocandidates. They broke down *S. aureus* biofilm more effectively than *P*. *aeruginosa* one. The highest biofilm inhibition activities were obtained 94.57 ± 4.99% and 89.28 ± 5.02% for nanoconjugate (**1b**) at 50 mg/L against S. *aureus* and *P. aeruginosa*, respectively. As the size of gold nanoparticles increased the antibiofilm activities diminished. Also, nanoconjugates (**1–3b**) displayed higher biofilm inhibition activities than nanoconjugates (**b**, **1–3**). Chatterjee et al. studied the antibiofilm activity of gold nanoparticles against *Vibrio cholera* biotypes. These nanoparticles inhibited the biofilm matrix formation at 100 μM.^62^ Sindelo et al. investigated the antibiofilm activities of morpholino-containing phthalocyanines against several microorganisms. The biofilm matrix formed by *S*. *aureus*, *E*. *coli*, *Klebsiella pneumoniae*, and *Salmonella choleraesuis* was inhibited significantly.^63^ Compared with the literature, biocandidates (**b**, **1–3**, and **1–3b**), especially the nanoconjugates (**1–3b**) can be utilized for biofilm inhibition applications.

**Figure 4:**
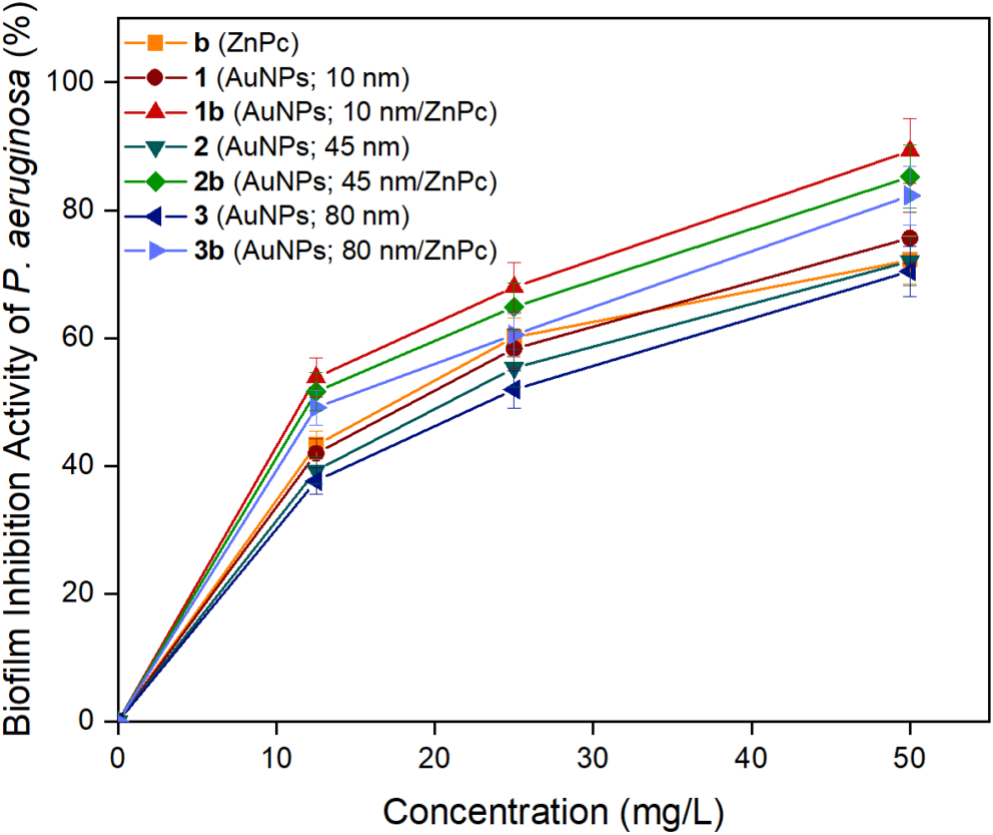
Biofilm inhibition activities of biocandidates **b** (ZnPc), **1** (AuNPs; 10 nm), **2** (AuNPs; 45 nm), **3** (AuNPs; 80 nm), **1b** (AuNPs; 10 nm/ZnPc), **2b** (AuNPs; 45 nm/ZnPc), and **3b** (AuNPs; 80 nm/ZnPc) against *S*. *aureus*.

**Figure 5:**
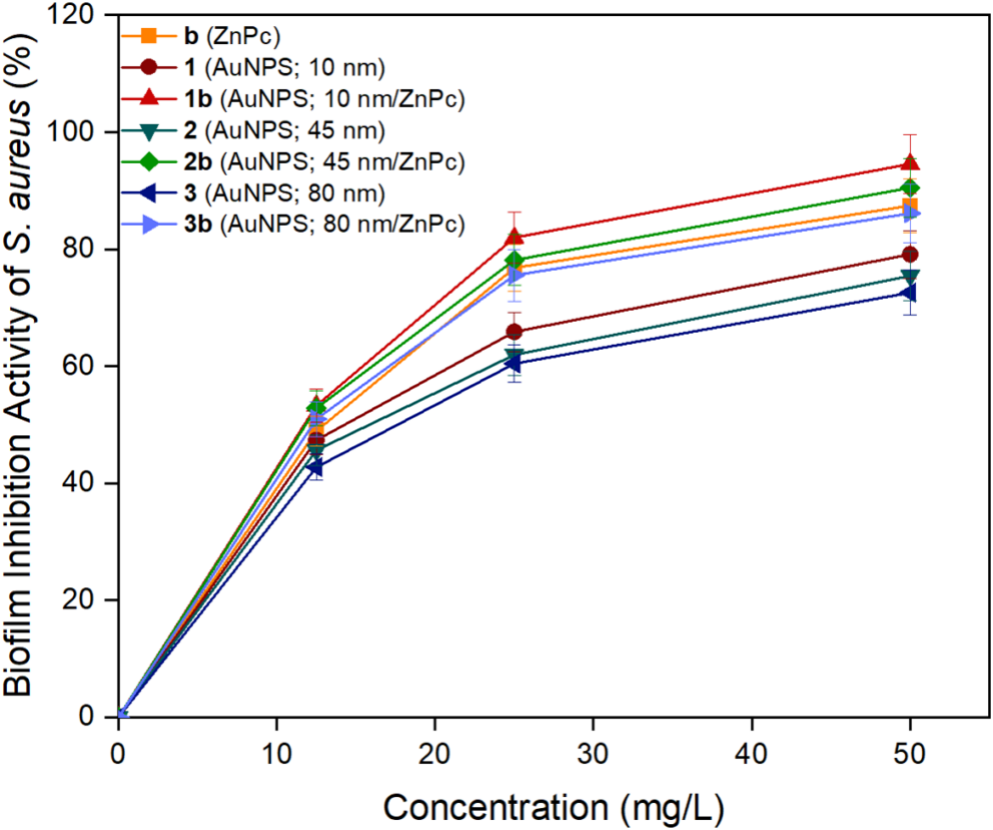
Biofilm inhibition activities of biocandidates **b** (ZnPc), **1** (AuNPs; 10 nm), **2** (AuNPs; 45 nm), **3** (AuNPs; 80 nm), **1b** (AuNPs; 10 nm/ZnPc), **2b** (AuNPs; 45 nm/ZnPc), and **3b** (AuNPs; 80 nm/ZnPc) against *P. Aeruginosa*.

### *E. coli* Viability Inhibition Activity

In this study, viability inhibition activities of biocandidates (**b**, **1–3**, and **1–3b**), released by diffusion, were examined against *E*. *coli* as a model microorganism. All the studied bioconjugates displayed high inhibition activities at different concentrations ranging from 12.5 to 50 mg/L (Figure 6). There was a direct correlation between increasing concentration and inhibition rate. The viability inhibition activities of biocandidates (**b**, **1–3**, and **1–3b**) were obtained 90.25 ± 5.48, 94.18 ± 5.85, 96.85 ± 5.62, 91.68 ± 5.74, 94.65 ± 5.76, 89.86 ± 5.81, and 93.15 ± 5.71% at 25 mg/L, respectively. Moreover, 100% inhibition activity was determined for all the studied biocandidates at 50 mg/L. Regardless of the size of gold nanoparticles, surficial modification of gold nanoparticles with macromolecule (**b**) refined the individual viability inhibition activities of gold nanoparticles and compound **b**. Farajzadeh et al. investigated viability inhibition activities of several new metal phthalocyanines bearing malonate groups at diverse concentrations (62.5–250 mg/L). Some complexes exhibited 100% inhibition activity at 250 mg/L concentration.^64^ Accordingly, biocandidates (**b**, **1–3**, and **1–3b**) can be clinically examined to ensure their potential as effective antimicrobial agents.

**Figure 6:**
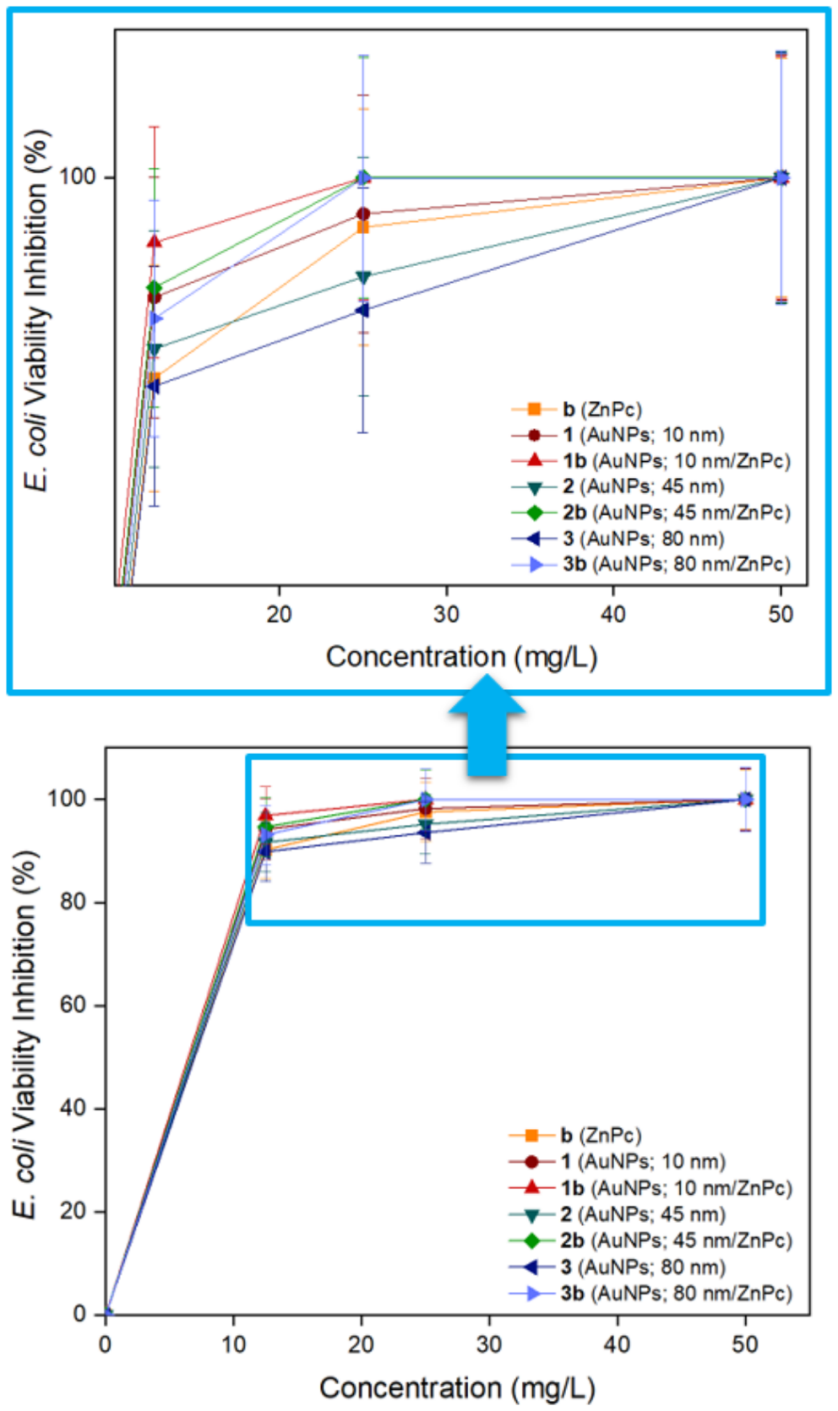
*E. coli* viability inhibition activities of biocandidates **b** (ZnPc), **1** (AuNPs; 10 nm), **2** (AuNPs; 45 nm), **3** (AuNPs; 80 nm), **1b** (AuNPs; 10 nm/ZnPc), **2b** (AuNPs; 45 nm/ZnPc), and **3b** (AuNPs; 80 nm/ZnPc).

### Antidiabetic Activity

Pancreatic amylases are responsible for hydrolysis of complex starch molecules in polysaccharide structure to maltose in disaccharide structure. Generally, the amount of postprandial glucose can decrease by inhibition or partial prevention of α-amylase activity during digestion which in turn can delay the hydrolysis, digestion, and absorption of carbohydrate. In this study, the antidiabetic properties of biocandidates (**b**, **1–3**, and **1–3b**) were investigated by measuring their α-amylase inhibition activities. The antidiabetic activities of biocandidates (**b**, **1–3**, and **1–3b**) are depicted in Figure 7. The respective antidiabetic activities of biocandidates (**b**, **1–3**, and **1–3b**) were obtained 54.78 ± 2.96, 13.71 ± 0.74, 51.06 ± 2.76, 11.06 ± 0.59, 46.09 ± 2.49, 7.52 ± 0.40, and 43.37 ± 2.34% at 100 mg/L. Macromolecule (**b**) and gold nanoparticles (**3**) exhibited the highest and lowest antidiabetic activities, respectively. An increase in the size of gold nanoparticles (**1–3**) was followed by a slight decrease in antidiabetic activity. However, their surficial functionalization with macromolecule (**b**) led to a considerable increase in antidiabetic activities by enhancing the size of gold nanoparticles. Some studies report the antidiabetic properties of several modified gold nanoparticles. Rafi et al. studied the antidiabetic properties of garcinol-functionalized gold nanoparticles. This nanostructure exhibited 50% inhibition activity at 8.9 μM.^65^ Kiran et al. examined the antidiabetic activities of gold nanoparticles synthesized from *Moringa oleifera*. As the concentration of gold nanoparticles increased, the enzyme activity decreased by approximately 50% inhibition at 130 mg/L.^66^ Günsel et al. investigated the antidiabetic potential of a new zinc­(II) phthalocyanine. The resultant compound displayed 50% enzyme inhibition values at the studied concentrations ranging from 0.95 to 1.93 μM.^55^ Compared to the literature, macromolecule **b** and nanoconjugates (**1–3b**) can be considered antidiabetic agents after further investigations.

**Figure 7:**
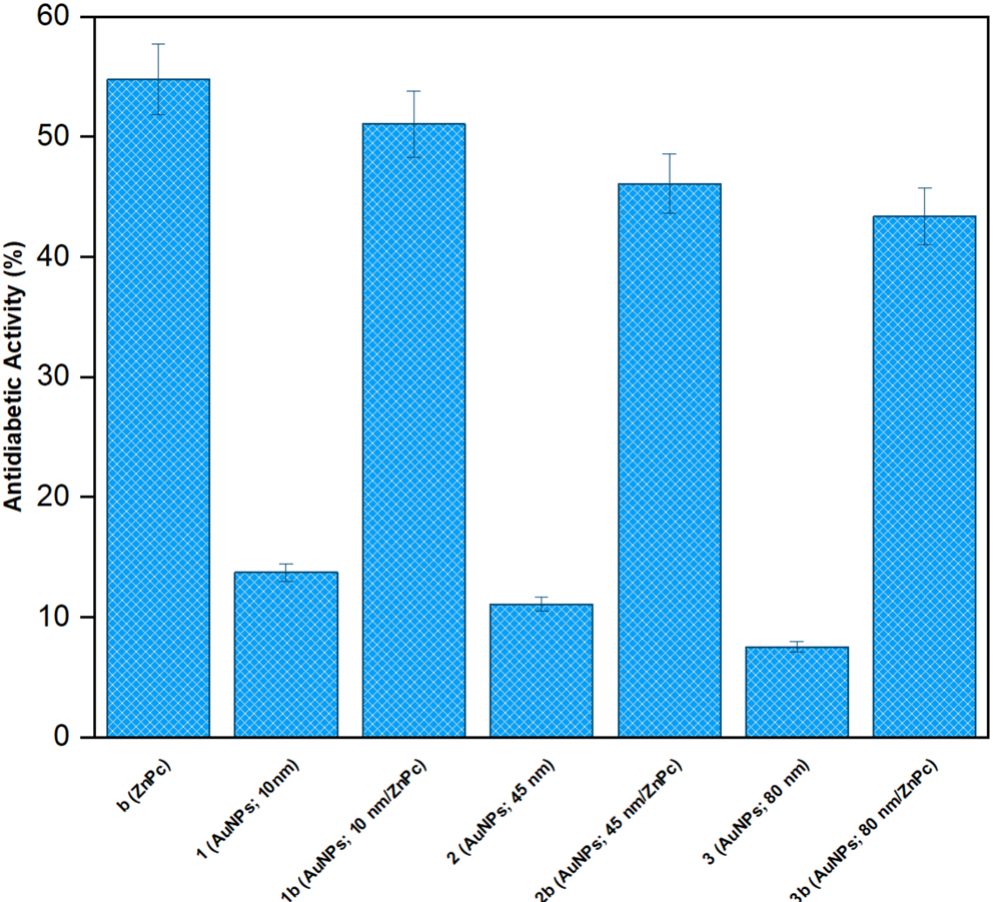
Antidiabetic Activities of of biocandidates **b** (ZnPc), **1** (AuNPs; 10 nm), **2** (AuNPs; 45 nm), **3** (AuNPs; 80 nm), **1b** (AuNPs; 10 nm/ZnPc), **2b** (AuNPs; 45 nm/ZnPc), and **3b** (AuNPs; 80 nm/ZnPc).

### DNA Cleavage Activity

The electrophoresis method is well-known as an appropriate approach for DNA separation, purification, and qualification studies. In this method, the release mechanism of biocandidates occurs by erosion and band analysis indicates accurate results can be applied to measure the effect of DNA cleavage materials on the superhelical form of DNA molecules. Indeed, this effect can lead to the formation of some bands via the conversion of Form I (superhelical) to Form II (nicked) and Form III (linear) as a result of single and/or double-chain breakages. The preservation of the native form (Form I) of the DNA molecule confirms no damage to the DNA molecule whereas the conversion of Form I (double strands) to a single strand (Form II) proves its cleavage by observation of a new band in agarose gel. The appearance of a new band (Form III) between Form I and Form II originates from cutting DNA molecules. In this study, the superhelical plasmid pBR 322 DNA cleavage activities of biocandidates (**b**, **1–3**, and **1–3b**) were investigated at different concentrations (25, 50, and 100 mg/L) using an agarose gel electrophoresis. The results are portrayed in Figure 8. Macromoleclue (**b**) resulted in single-chain breakage of DNA molecules at all the studied concentrations whereas gold nanoparticle (**1**) led to the single-chain breakage (at 25 mg/L) and double-chain breakage (at 50 and 100 mg/L). Nanoconjugate (**1b**) induced single-chain breakage at 25 mg/L, double-chain breakage at 50 mg/L, and complete disintegration at 100 mg/L. Besides, biocandidates (**2**,**2b**, and **3**) resulted in a single strand break at 25 mg/L while they led to complete fragmentation of the DNA molecule at 50 mg/L and 100 mg/L. Biocandidates (**3b**) caused single-strand breakage at 25 mg/L and double-strand breakage at 50 and 100 mg/L. The size of gold nanoparticles (**1–3**) did not affect the DNA cutting efficiency at 25 mg/L. However, gold nanoparticles (**2** and **3**) completely degraded DNA molecules at 50 and 100 mg/L. The obtained results were compared to the findings of some studies in the literature. Amitha and Vasudevan investigated the DNA shearing activities of some zinc­(II) phthalocyanines at different concentrations. All the studied complexes exhibited good DNA cleavage activities.^67^ Huang et al. indicated that the methylation morpholine-phthalocyanine@gold nanorods displayed high DNA cleavage activities.^68^ Accordingly, biocandidates (**b**, **1–3**, and **1–3b**) demonstrated high DNA fragmentation activities that can attract attention as multidisciplinary agents to design efficient clinical pharmaceutical materials.

**Figure 8:**
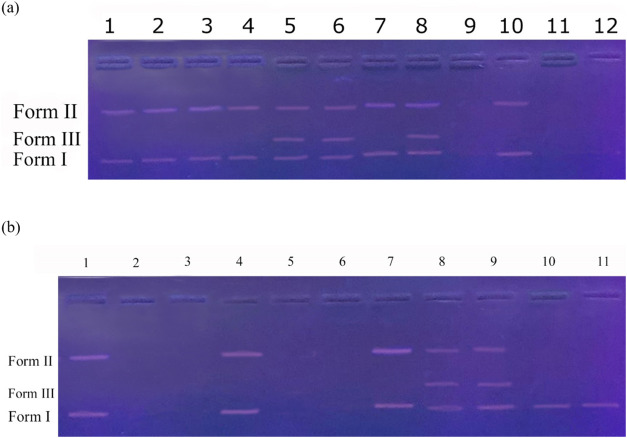
DNA cleavage activity: (a) 1:25 mg/L of **b** + pBR 322 DNA; 2:50 mg/L of **b** + pBR 322 DNA; 3:100 mg/L of **b** + pBR 322 DNA; 4:25 mg/L of **1** + pBR 322 DNA; 5:50 mg/L of **1** + pBR 322 DNA; 6:100 mg/L of **1** + pBR 322 DNA; 7:25 mg/L of **1b** + pBR 322 DNA; 8:50 mg/L of **1b** + pBR 322 DNA; 9:100 mg/L of **1b** + pBR 322 DNA; 10:25 mg/L of **2** + pBR 322 DNA; 11:50 mg/L of **2** + pBR 322 DNA; 12:100 mg/L of **2** + pBR 322 DNA; (b) 1:25 mg/L of **2b** + pBR 322 DNA; 2:50 mg/L of **2b** + pBR 322 DNA; 3:100 mg/L of **2b** + pBR 322 DNA; 4:25 mg/L of **3** + pBR 322 DNA; 5:50 mg/L of **3** + pBR 322 DNA; 6:100 mg/L of **3** + pBR 322 DNA;7:25 mg/L of **3b** + pBR 322 DNA;8:50 mg/L of **3b** + pBR 322 DNA;9:100 mg/L of **3b** + pBR 322 DNA; 10: pBR 322 DNA; 11: DMSO + pBR 322 DNA.

## Experimental Section

### Materials

4,5-dichlorophthalonitrile (99.0%), 4-phenylthiazole-2-thiol (90.0%), potassium carbonate (≥99.0%), dimethyl sulfoxide (≥99.9%), ethanol (pure, ≥ 99.5%), zinc­(II) acetate (90.9%), chloroauric acid (90.9%), trisodium citrate dihydrate, *n*-hexane (≥99.0%), and tetrahyrofuran (≥99.9%) were purchased from Sigma-Aldrich, Germany.

### Synthesis and Characterization

#### Compound **a**

4,5-dichlorophthalonitrile (1 g, 5.08 mmol), 4-phenylthiazole-2-thiol (2 g, 10.35 mmol), and potassium carbonate (1 g, 7.61 mmol) were stirred in dry dimethyl sulfoxide at 80 °C under an inert atmosphere for 48 h. The reaction content was poured into an iced water mixture and stirred for 1 h. The precipitation was filtered off, dried, and purified by recrystallization from ethanol. Chemical Formula: C_26_H_14_N_4_S_4_. Yield 1.75 g (67.5%). FT-IR υ (cm^–1^): 3104 (aromatic CH), 2230 (C≡N), 1072 (C–S–C). ^1^H NMR (500 MHz; DMSO-*d*_6_): δ (ppm) 8.35 (s, 2H), 8.31 (s, 2H), 7.97–7.91 (d, 4H), 7.49–7.42 (t, 4H), 7.40–7.34 (t, 2H). ^13^C NMR (500 MHz; DMSO-*d*_6_): δ (ppm) 157.22; 156.39; 142.01; 136.51; 133.68; 129.40; 129.08; 126.55; 119.84; 115.52; 115,47.

#### Compound **b**

Compound **a** (0.100 g, 0.196 mmol) and anhydrous zinc­(II) acetate (0.006 g, 0.049 mmol) were stirred in *N*, *N*-dimethylaminoethanol at 135 °C under a nitrogen atmosphere for 24 h. The crude content was treated with a mixture of ice/water and stirred for 1 h. The precipitation was filtered off and dried. The pure product was obtained using a chromatographic approach on silica gel eluted with a mixture of *n*-hexane: tetrahydrofuran (5:1). Chemical Formula: C_104_H_56_N_16_S_16_Zn. Yield 53 mg (51%), mp >250 °C. FT-IR υ (cm^–1^): 3071 (aromatic CH), 1067 (C–S–C). ^1^H NMR (500 MHz; DMSO-*d*_6_): δ (ppm) 8.35 (s, 8H), 8.31 (s, 8H), 7.96–7.92 (d, 16H), 7.49–7.43 (t, 16H), 7.40–7.35 (t, 8H). MS (MALDI-TOF): *m*/*z* calcd. for [M]^+^ 2108.08 found 1910.49 [M-2C_9_H_6_NS_2_-2H+DIT]^+^. UV–vis (THF): λ_max_ nm (log ε) 365 (4.50), 713 (5.16).

#### Gold Nanoparticles (**1–3**)

Three different groups of gold nanoparticles differing in size were prepared by changing the chloroauric acid/trisodium citrate ratio. Briefly, the aqueous solution of chloroauric acid was heated to the boiling point. Then, trisodium citrate solution (7 mL for **1**; 4 mL for **2**, and 1.5 mL for **3**) was added and the mixture was stirred for 15 min.^19^

#### Nanoconjugates (**1–3b**)

Ten mg macromolecule (**b**) was dissolved in an adequate amount of dimethyl sulfoxide and added to 10 mL of the prepared gold nanoparticles (**1–3**). The mixture was stirred at room temperature for 10 h, centrifuged, and recollected.^19^

### Biological Studies

#### DPPH Radical Scavenging Ability

The free radical scavenging activities of biocandidates (**b**, **1–3**, and **1–3b**) were determined at different concentrations ranging from 6.25 to 100 mg/L by applying the DPPH• free radical assay. A 0.004% DPPH solution (1000 μL) was added to each of the samples (250 μL) including different concentrations of biocandidates (**b**, **1–3**, and **1–3b**) and incubated for 30 min at room temperature in the dark. The absorbance was measured at 517 nm using a spectrophotometer. The sample containing only DPPH free radical was used as a control. The antioxidant activity was calculated by applying eq 1

$$\mathrm{an}tioxidant\;activity\;(\%)=({Abs}_{\left(control\right)}-{Abs}_{\left(sample\right)}/{Abs}_{\left(control\right)})\times 100$$ 1

### Antimicrobial and Antimicrobial Photodynamic Therapy Activity

Antimicrobial and antimicrobial photodynamic therapy activities of biocandidates (**b**, **1–3**, and **1–3b**) were studied against several microorganisms by performing the broth microdilution method. Serial dilution of biocandidates (**b**, **1–3**, and **1–3b**; 1024, 512, 256, 128, 64, 32, 16, 8, 4, 2, and 1 mg/L) was performed using Nutrient Broth (Merck) to a final volume of 150 μL in 96-well plates. Fifteen μL of the target microorganism suspension (2 × 10^8^ CFU/mL) was added to each plate well. The plate wells were incubated for 24 h at 37 ± 0.1 °C and the minimum inhibition concentration (MIC) values were calculated. The antimicrobial activities were performed in triplicate. In addition, the same process was applied to APDT studies with some modifications. The antimicrobial activities of biocandidates (**b**, **1–3**, and **1–3b**) were considered after their exposure to light for 30 min. A red-orange light-emitting diode was used at λ 632 ± 2 nm with 12 J/cm^2^ energy.

### Biofilm Inhibition Activity

The biofilm inhibition activities of biocandidates (**b**, **1–3**, and **1–3b**) were examined against *P. aeruginosa* and *S. aureus* bacteria. Each microorganism was added to well plates containing different concentrations of biocandidates (**b**, **1–3**, and **1–3b**; 0, 12.5, 25, and 50 mg/L). The well plates were incubated 72 h at 37 °C and then carefully drained, and washed twice with distilled water. The formed biofilm was treated with crystal violet (CV) for 1 h. After removing the residual CV, the plates were gently rinsed with distilled water. Then, the plates were treated with ethanol for 15 min for CV recovery to spectrophotometrically determine the amount of CV absorbed by the biofilm material. A spectrophotometer was performed to measure the biofilm inhibition at 595 nm. The biofilm formation inhibition was determined by applying eq 2

$$b\mathrm{iofilm}\;i\mathrm{nhibition}\;\left(\%\right)=\left({A\mathrm{bs}}_{\left(\mathrm{control}\right)}-{A\mathrm{bs}}_{\left(\mathrm{sample}\right)}/{A\mathrm{bs}}_{\left(\mathrm{control}\right)}\right)\times 100$$ 2

### Microbial Cell Viability Test

The biological activities of biocandidates (**b**, **1–3**, and **1–3b**) capacity to inhibit bacterial cell viability of *E. coli* (ATCC 25922) were investigated in this study, as well. *E. coli* was inoculated into Nutrient Broth (NB) and grown at 37.0 °C for 24 h in a shaker. After 1 day-incubation, *E. coli* was separated by centrifugation at 5500 rpm for 6 min. The residue of the NB medium was then rinsed off the bacterial pellet with a sterile 0.9% saline solution. The microbial suspension (2.8 × 109 CFU/mL) was obtained by the addition of the *E. coli* strain to saline (10 mL) and used for the microbial cell viability experiments. *E. coli* was treated with different concentrations of biocandidates (**b**, **1–3**, and **1–3b**; 0, 12.5, 25, and 50 mg/L) at 37 °C for 2 h. The mixtures were then diluted with a variety of ratios, inoculated on NB agar, and left to incubate for 24 h at 37 °C. The colonies were counted and the related microbial cell was calculated by applying eq 3

$$m\mathrm{icrobial}\;\mathrm{cell}\;\mathrm{viability}\;inhibition\;\left(\%\right)=\left(A_{\left(\mathrm{control}\right)}-A_{\left(\mathrm{sample}\right)}/A_{\left(\mathrm{control}\right)}\right)\times 100$$ 3

### Antidiabetic Activity

The antidiabetic activities of biocandidates (**b**, **1–3**, and **1–3b**) were determined at 100 mg/L. Each biocandidate (**b**, **1–3**, and **1–3b**) was incubated with α-amylase enzyme for 10 min at 37 °C. Then, Starch was added to the test content and incubated again for 20 min at 37 °C. After the addition of 3,5 dinitro salicylic acid (3.5 DNS), the content was boiled for 5 min at 100 °C. The test content was cooled to room temperature and diluted. The spectrophotometric measurements were carried out at 540 nm. The content excluding the test biocandidates was utilized as a control. The antidiabetic activity was calculated by performing eq 4

$$\mathrm{antidiabetic}\;\mathrm{activity}\;(\%)=({A\mathrm{bs}}_{\left(\mathrm{control}\right)}-{A\mathrm{bs}}_{\left(\mathrm{sample}\right)}/{A\mathrm{bs}}_{\left(\mathrm{control}\right)})\times 100$$ 4

### DNA Cleavage Activity

The DNA cleavage activities of biocandidates (**b**, **1–3**, and **1–3b**) were studied at three different concentrations (25, 50, and 100 mg/L). Different concentrations of the biocandidates (15 μL) were added to PCR tubes containing pBR 322 plasmid DNA (5 μL) and incubated for 2 h at 37 °C in the dark. Agarose gel (1%) was placed in the electrophoresis tank. The gel was fixed and TAE buffer was added to the tank. After loading dye on each biocandidate (**b**, **1–3**, and **1–3b**), the samples were loaded into the gel wells. And the (+) and (−) ends of the electrophoresis tank were connected. The electrical power device was set to 100 V (V) for 60 min. Then, the gel was gently removed from the electrophoresis tank and imaged using a UV transilluminator.

## Conclusions

In this study, a new disubstituted phthalonitrile and its zinc­(II) phthalocyanine were synthesized and utilized as a modifying group to alter three different sizes of gold nanoparticles. The bioactivity of all the unmodified and modified gold nanoparticles was investigated by studying extensively their diverse biological properties. As compared, the nanoconjugates exhibited much higher biological activities than the unmodified gold nanoparticles. These results proved the improving effect of the thiazole-containing zinc­(II) phthalocyanine on the modified gold nanoparticles. Additionally, the exhibition of the highest biological activities for the smallest functionalized gold nanoparticles confirmed the efficient influence of the high surface-to-volume ratio on the unique features of the nanostructures. Interestingly, the phthalocyanine-modified gold nanoconjugates displayed higher *E*. *coli* viability inhibition activities than the zinc­(II) phthalocyanines and unmodified gold nanoparticles. The obtained results indicated that the zinc­(II) phthalocyanine and gold nanoparticles can improve their individual characteristics. Also, the antimicrobial activities (especially APDT activities) of all the bioagents increased with irradiation. Most of the tested bioagents exhibited high DNA cleavage activities by cutting the DNA molecule from both strands, however, some of them split the DNA molecule to its nucleotides. To conclude, the prepared phthalocyanine-gold nanoconjugates can be considered potential therapeutic agents for many biological applications after further research. Moreover, this study can hopefully fill the vacancy for scientific research on the findings of efficient phthalocyanine-based nanomaterials for multidisciplinary biological applications.

## Supplementary Material



## Data Availability

The data that support the findings of this study are available throughout the manuscript and supporting files.

## Notes

^13^C NMR spectrum of compound **a**, ^1^HNMR spectra of compound (**a** and **b**), FTIR spectra of compounds (**a** and **b**), MALDI-TOF spectrum of compound **b**, UV–vis spectrum of compound **b**, FTIR spectra of nanostructures (**1** and **1b**) (PDF)
