# Supplementary material for: Biological Effect of Thiazole-Containing Zinc(II) Phthalocyanine on Different Sizes of Gold Nanoparticles
Source: ACS Omega. 2025 Jul 3;10(27):28709–20. doi: 10.1021/acsomega.4c08762 (PMC12268402; doi:10.1021/acsomega.4c08762)
Supplement: Supplementary file 1 [file ao4c08762_si_001.pdf]

Biological effect of thiazole-containing zinc (II) phthalocyanine on different sizes of gold nanoparticles

Nazlı Farajzadeh Öztürk,[a] Hilal Zengin Uzunmehmetoğlu,[b] Hacer Yasemin Yenilmez,[b] Sadin Özdemir,[c] Abdurrahman Dündar,[d] Zehra Altuntaş Bayır*[b]

[a]*Faculty of Pharmacy, Department of Analytical Chemistry, Acıbadem Mehmet Ali Aydınlar University, Atasehir, TR-34752, Istanbul, Türkiye*

[b]*Department of Chemistry, Istanbul Technical University, Maslak, TR-34469, Istanbul, Türkiye*

[c]*Food Processing Programme, Technical Science Vocational School, Mersin University, TR-33343 Yenisehir, Mersin, Türkiye*

[d]*Department of Medical Services and Techniques, Vocational School of Health Services, Mardin Artuklu University, TR-47420, Mardin, Türkiye*

Corresponding author e-mail address: bayir@itu.edu.tr (Prof. Dr. Zehra Altuntaş Bayır)

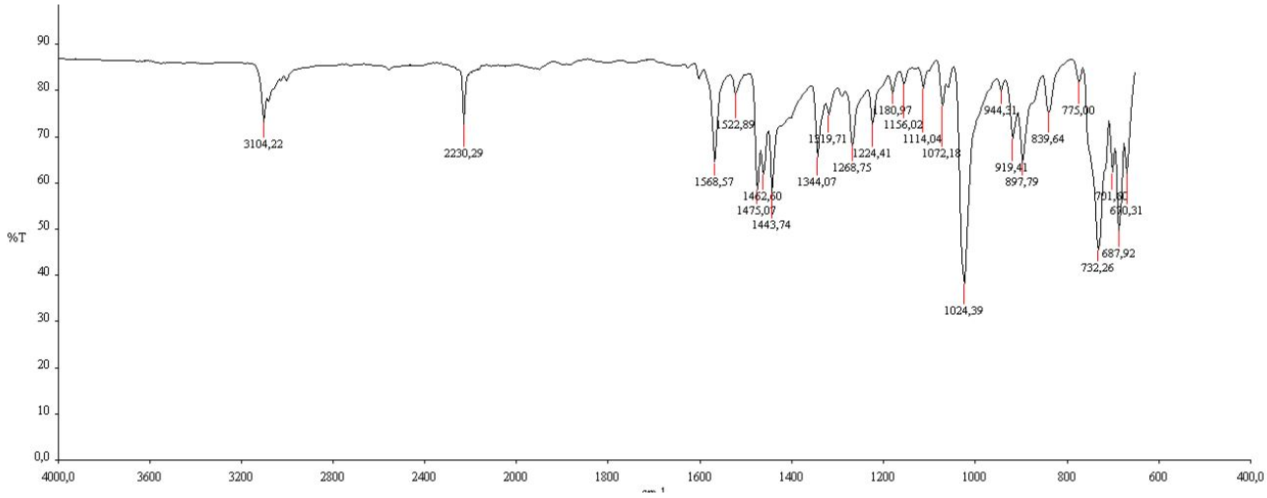

S1. FT-IR spectrum of compound a.

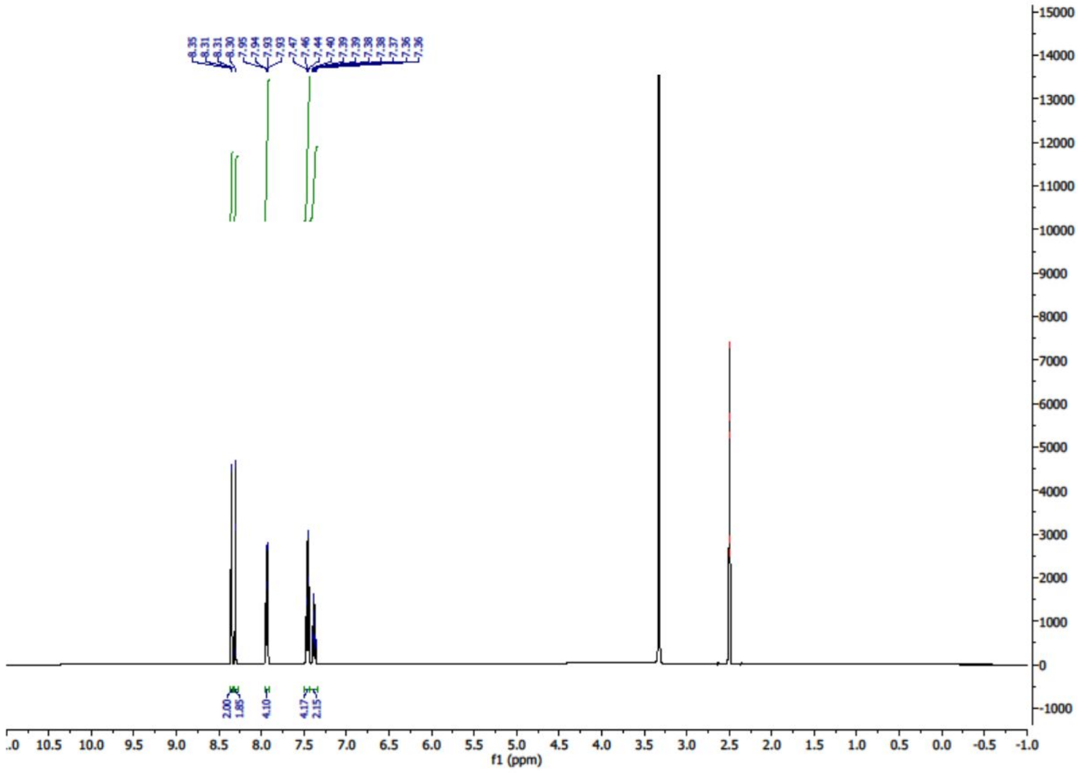

S2. ^{1}H NMR spectrum of compound a.

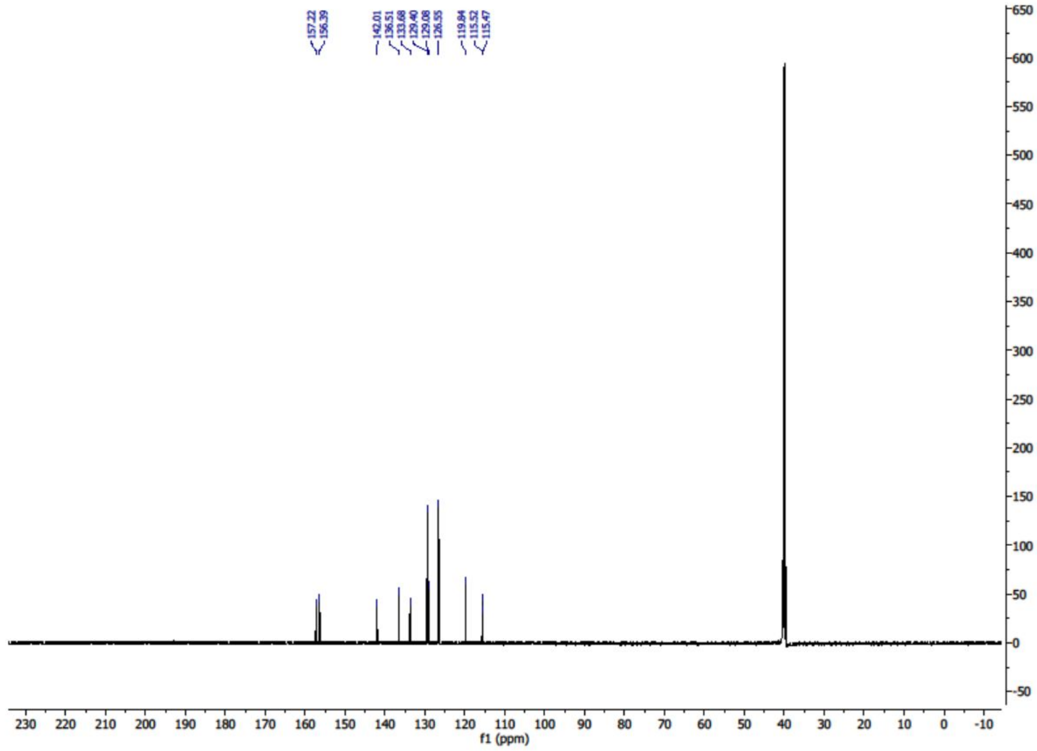

S3. ^{13}C NMR spectrum of compound a.

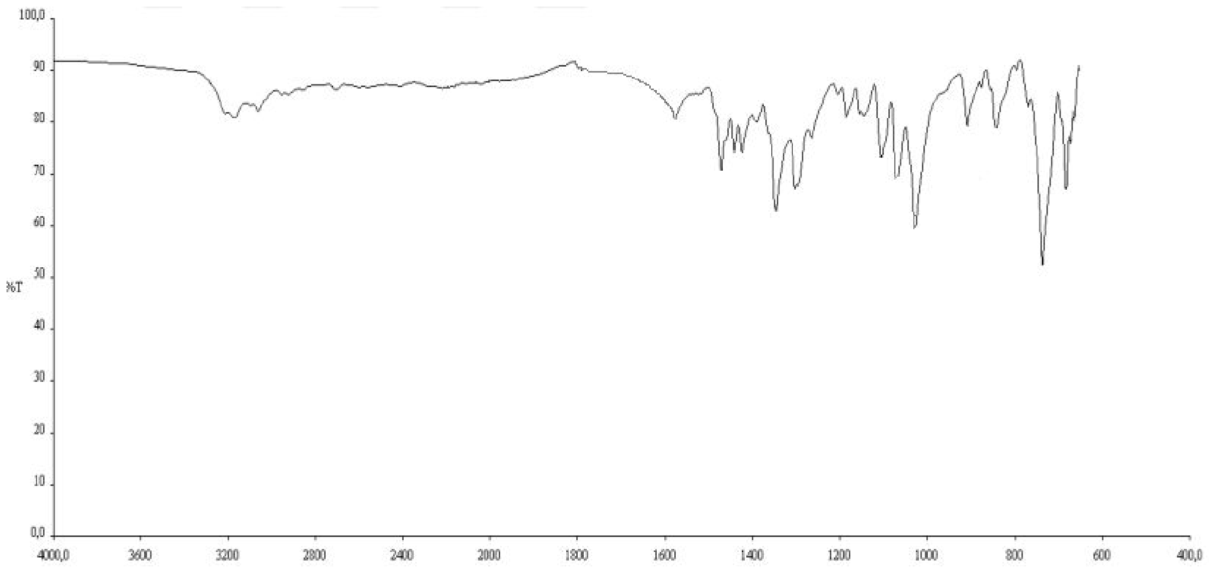

S4. FT-IR spectrum of compound b.

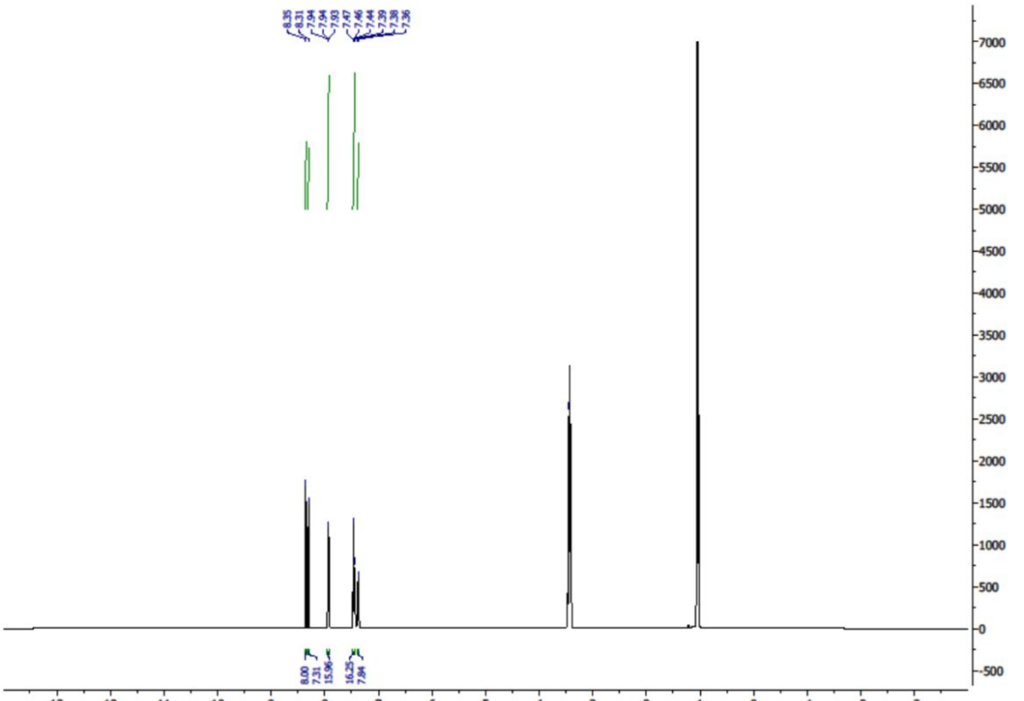

S5. ^{1}H NMR spectrum of compound b.

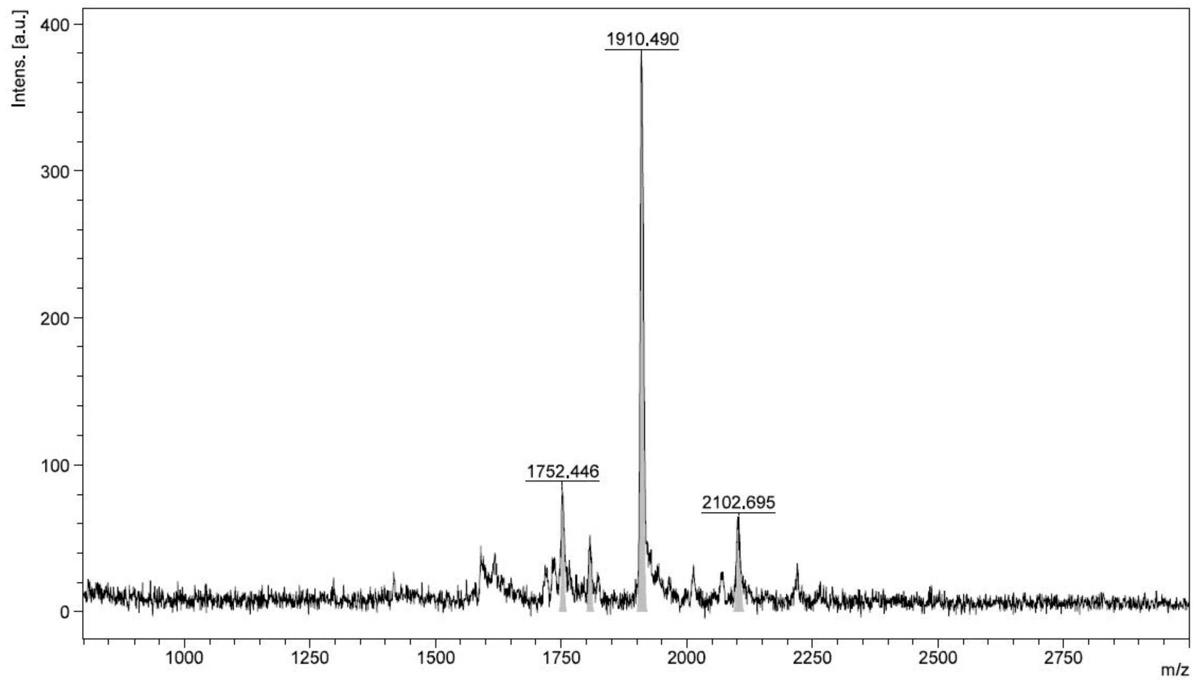

S6. MALDI-TOF spectrum of compound b.

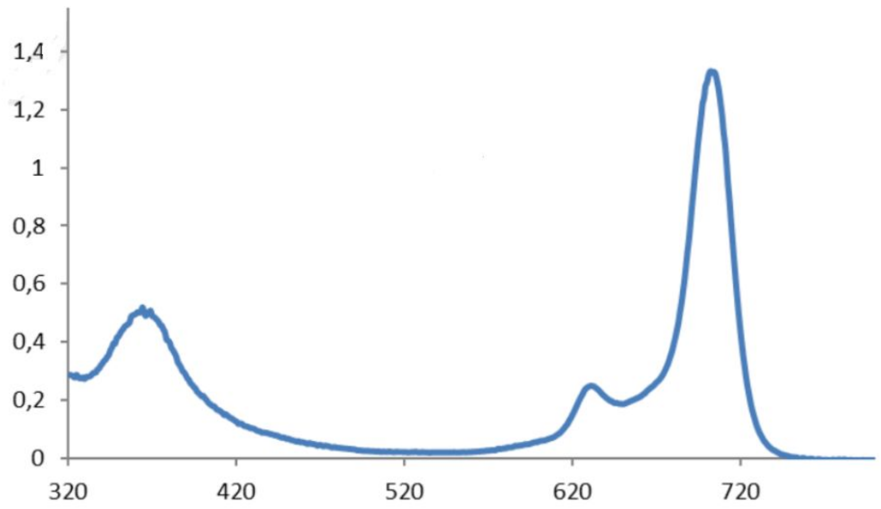

S7. UV-vis spectrum of compound b.

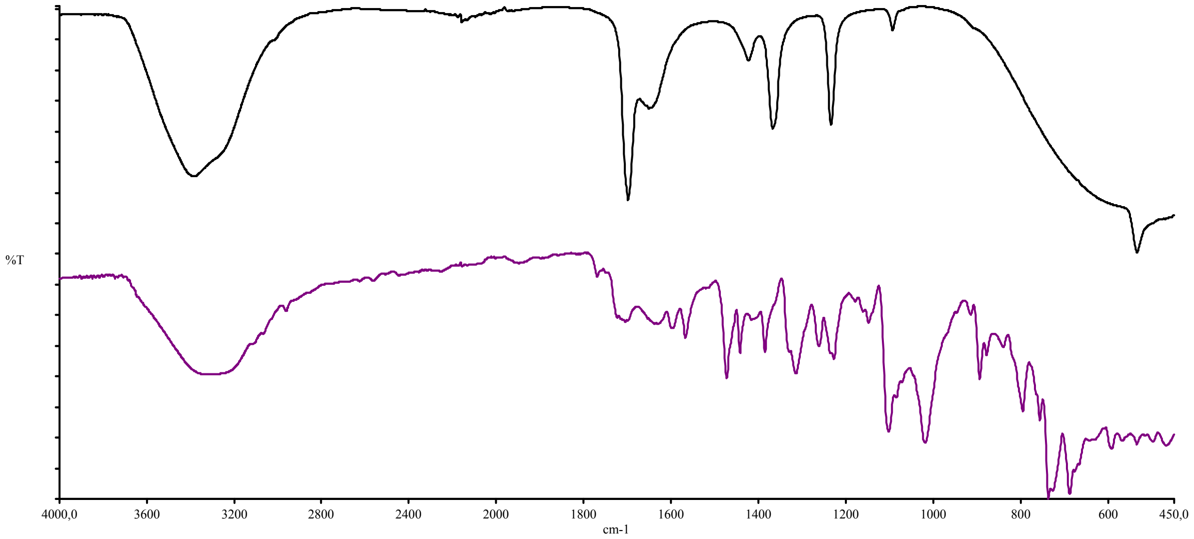

S8. FT-IR spectra of gold nanoparticles (1; black line) and nanoconjugate (1b: purple).
